# Time and Financial Costs for Physician Practices to Participate in the Medicare Merit-based Incentive Payment System: A Qualitative Study

**DOI:** 10.1001/jamahealthforum.2021.0527

**Published:** 2021-05-14

**Authors:** Dhruv Khullar, Amelia M. Bond, Eloise May O’Donnell, Yuting Qian, David N. Gans, Lawrence P. Casalino

**Affiliations:** 1 Division of Health Policy and Economics, Department of Population Health Sciences, Weill Cornell Medical College, New York, New York; 2 Division of General Internal Medicine, Department of Medicine, Weill Cornell Medical College, New York, New York; 3 Medical Group Management Association, Englewood, Colorado

## Abstract

**Importance:**

The Merit-based Incentive Payment System (MIPS) is a major Medicare value-based purchasing program, influencing payment for more than 1 million clinicians annually. There is a growing concern that MIPS increases administrative burden, and little is known about what it costs physician practices to participate in the program.

**Objective:**

To examine the costs for independent physician practices to participate in MIPS in 2019.

**Design, Setting, and Participants:**

This qualitative study identified and interviewed leaders of physician practices participating in the US Centers for Medicare & Medicaid Services (CMS) MIPS program, including those in MIPS alternative payment models. Time required and financial costs were calculated from responses to in-depth, semistructured interviews conducted from December 12, 2019, to June 23, 2020. Physician practices were categorized by size (small, 1-9 physicians; medium, 10-25; and large, ≥50), specialty (primary care, general surgery, or multispecialty), and US census region. Participants were asked about 2019 costs related to clinician and staff time, information technology, and external vendors. Time was converted to financial costs using the Medical Group Management Association’s Provider Compensation and the Management and Staff Compensation databases.

**Main Outcomes and Measures:**

Annual time spent by staff on MIPS-related activities and mean per-physician costs to physician practices in 2019.

**Results:**

Leaders of 30 physician practices (9 [30.0%] small primary care, 6 [20.0%] small general surgery, 4 [13.3%] medium primary care, 4 [13.3%] medium general surgery, and 7 [23.3%] large multispecialty) represented all US census regions, and 14 of the 30 (46.7%) practices participated in a MIPS alternative payment model in 2019. The mean per-physician cost to practices of participating in MIPS was $12 811 (interquartile range [IQR], $2861-$17 715). Physicians, clinical staff, and administrative staff together spent 201.7 (IQR, 50.9-295.2) hours annually per physician on MIPS-related activities. Medical assistants and nursing staff together spent a mean of 99.2 (IQR, 0-163.3) hours per physician each year; frontline physicians spent 53.6 (IQR, 0.6-55.8) hours; executive administrators spent 28.6 (IQR, 3.1-26.7) hours; other clinicians and staff spent a combined 20.3 (IQR, 0-36.8) hours. Physician time accounted for the greatest proportion of overall MIPS-related costs (54%; $6909; IQR, $94-$9905).

**Conclusions and Relevance:**

In this qualitative study, physician practice leaders reported significant time and financial costs of participating in the MIPS program. Attention to reducing the burden of MIPS may be warranted.

## Key Points

**Question:**

How much does it cost for physician practices to participate in Medicare’s Merit-based Incentive Payment System (MIPS)?

**Findings:**

In this qualitative study using interviews with leaders of 30 physician practices across the US, an average of $12 811 per physician was spent to participate in MIPS in 2019. Clinicians and administrators spent more than 200 hours per physician on MIPS-related activities.

**Meaning:**

The time and financial costs of MIPS participation are considerable; policy makers should consider ways to reduce the administrative and financial burden of MIPS participation for physician practices.

## Teaser

This qualitative study interprets information collected through interviews with leaders of 30 physician practices across the US to assesses the time and financial costs of participating in the Medicare Merit-based Incentive Payment System.

## Introduction

The Merit-based Incentive Payment System (MIPS) is a major value-based purchasing program under the Medicare Access and CHIP Reauthorization Act,^1^ which aims to hold health care clinicians and health care organizations accountable for the quality and cost of care and influences payment for about 1 million clinicians each year, including more than 300 000 physicians. Through the Act, clinicians can choose to participate in 1 of 2 tracks: (1) advanced alternative payment models (A-APMs), which include risk-based bundled payment programs, advanced patient-centered medical homes, and some accountable care organizations, or (2) MIPS, which reimburses clinicians largely through fee-for-service payments that adjust based on performance in the program. Within MIPS, clinicians can choose to participate through the default pathway or an alternative payment model (APM), similar to the A-APMs, but without the downside financial risk.^2^ In both MIPS tracks, performance is evaluated in 4 domains: quality, improvement activities, promoting interoperability, and costs.

The MIPS program is designed to be budget-neutral, meaning that the funds available for rewarding superior performance cannot exceed the penalties imposed on clinicians with poor performance. An exception to this requirement is a $500 million pool available to reward clinicians who meet an exceptional performance threshold. Because many more clinicians have received high scores than low scores, the program’s rewards have been modest to date. In 2020, for example, clinicians with a perfect MIPS score received a maximum positive payment adjustment of 1.68%, based on 2018 performance.^3^ Some clinicians and health care leaders have argued that these small incentives are insufficient to cover the costs of participating in MIPS.^4^ Clinicians participating in A-APMs, by contrast, receive a 5% lump-sum bonus based on the previous year’s Medicare Part B (medical, not hospital services) payments.^5^ Clinicians are exempt from MIPS if they do not meet low-volume thresholds for Part B services; that is, billing at least $90 000 in services, seeing more than 200 patients, and providing at least 200 professional services to patients.^6^

It remains unclear whether MIPS accurately captures quality or effectively incentivizes improvements in health care delivery, but there is a growing concern that the program increases administrative burden for clinicians and medical practices.^7,8,9,10,11^ A recent survey of more than 400 physician practices found that 76% of respondents felt that MIPS is very or extremely burdensome, and 87% report that MIPS payment adjustments do not cover the cost of time and resources needed for program participation.^12^ The actual participation cost to physician practices is not known, but prior research on incentive programs^13,14^ suggests these costs can be considerably high, and that small practices, in particular, may not have the resources needed to succeed. For example, a 2016 study^13^ found that physician practices spend more than $40 000 per physician annually on quality reporting activities for various insurers. More generally, physician practices report spending more time dealing with external quality programs in recent years.^7,10,12^

To our knowledge, no published research has examined the costs incurred by physician practices to participate in the MIPS program. We conducted a series of in-depth, semistructured interviews to calculate the financial and time costs for primary care, general surgery, and large multispecialty physician practices participating in MIPS in 2019.

## Methods

### Setting, Participants, and Study Design

We conducted semistructured 45- to 60-minute telephone interviews with physician practice leaders across the US to understand their costs related to participating in MIPS. While conducting a survey would have made it possible to base the estimates on a larger sample of practices, we believed that because of the complexity of MIPS, interviews would more accurately identify costs.

Practices and administrative contacts were identified using the Medical Group Management Association’s membership database.^15^ Practices were categorized by specialty, size, and census region. Primary care and general surgery practices were defined as having at least 80% of physicians practicing in its dominant specialty; multispecialty practices were self-identified as having multiple primary care, medical, and surgical specialties (eMethods 1 in the Supplement). Small practices were defined as having 1 to 9 physicians; medium practices, 10 to 25 physicians; and large practices, 50 or more physicians.

Potential participants were randomly selected within size, specialty, and region categories, and then invited via email message. Practices that did not respond were sent 2 follow-up email messages and received 2 follow-up telephone calls. Prior to the interview, each practice received a screening questionnaire that confirmed MIPS participation, the number and specialty of physicians, and whether the practice participated in a MIPS A-APM in 2019. We distinguished non–APM vs APM practices because prior research suggests^16,17^ that they may have different reporting MIPS infrastructure and performance; thus, MIPS-related participation costs also may vary.

In total, 185 physician practices were invited, and 30 practices participated in interviews. Interviews were conducted with physician executives or senior nonphysician administrators. All interviews were conducted by at least 1 faculty member (D.K., L.P.C., A.M.B., or D.N.G.) and occurred from December 2019 through June 2020; a research assistant took notes.

 The Institutional Review Board at Weill Cornell Medical College waived review and informed consent because the study was determined to meet exemption requirements per 45 CFR §46.104(d). Leaders of small practices were offered a $1000 to $1500 incentive to participate; those at medium and large practices were offered $500. Findings are presented in adherence to the Standards for Reporting Qualitative Research (SRQR) reporting guideline.

### Interview Protocol

The interview protocol was developed through extensive review of literature^2,7,9,16,18^ on MIPS and other pay-for-performance programs; articles that used surveys to estimate health plan and quality-reports costs to physician practices^13,19,20^; and pilot interviews with physicians and quality-reporting experts. The interview instrument (eDocuments 1 and 2 in the Supplement) comprised questions about the time and financial costs associated with MIPS participation for 2019, including those related to staff time, information technology (IT), and external consultants and vendors. Throughout each interview, participants were repeatedly and explicitly asked whether a given cost was associated with MIPS specifically or more generally with quality reporting. Each physician practice also reported the proportion of patients insured by Medicare at its practice or the percentage of overall revenue it received from Medicare.

### Data Analysis

#### Interview Coding

Three research team members reviewed each interview transcript (D.K., E.M.O., and Y.Q.) and independently coded 10 interviews for the costs reported. Given the greater than 95% concordance, the remaining transcripts were coded by E.M.O. and Y.Q. with guidance from D.K. Any remaining issues were brought to the entire research team for resolution. Microsoft Word and Excel, versions 16.0 (Microsoft Corp) were used to manage the data.

#### Separating Costs of MIPS From Costs of Other Quality Programs

In some cases, it was difficult for physician practice leaders to disaggregate costs of participating in MIPS from costs of other quality-reporting programs. In such cases, we used a practice’s percentage of Medicare fee-for-service patients or Medicare fee-for-service revenue to scale costs. For example, if a practice purchased a software package to participate in multiple quality-reporting programs and 20% of its revenue came from Medicare fee-for-service, we ascribed 20% of that cost to MIPS participation. When only overall Medicare (ie, including Medicare Advantage) percentages were provided, we approximated the Medicare fee-for-service percentage by subtracting the Medicare Advantage rate in the practice’s county.^21^ When ambiguity existed around the number of hours or the cost of resources spent on MIPS-related activities, we used the most conservative (lowest) estimate.

#### Calculating Costs

We calculated total costs by adding the costs of IT and external vendors used for MIPS to the costs of MIPS-related time spent by the practice’s staff. We asked practice leaders for the time spent on activities and used their answers to calculate hours per year spent by practice physicians and staff, including time spent tracking quality measures, attending training sessions, creating or implementing quality improvement activities, and collecting quality data and/or entering information into patients’ electronic health record (EHR). Time spent on MIPS-related activities was recorded for (1) physicians, (2) physician leaders, (3) advanced practice practitioners (nurse practitioners and physician assistants), (4) nursing staff (licensed practical nurses [LPNs], medical assistants [MAs], and registered nurses [RNs]), (5) executive administrators, and (6) other administrative staff. Physician-leader time was defined as additional time they spent on MIPS-related activities compared with other practicing “rank-and-file” physicians.

We converted clinician and administrator time into dollars using annual median compensation and benefits reported in the Medical Group Management Association (MGMA) Provider Compensation and Management and Staff Compensation databases.^22,23^ Per recent MGMA data,^22^ physicians were assumed to work 51.4 hours per week and 48 weeks per year. Nonphysicians were assumed to work 40 hours per week, 48 weeks per year for nonexempt (eg, nurses, receptionists, billing staff, coders) and 47 weeks per year for exempt (eg, administrative) staff.

The costs for IT included the purchase of new EHR or software packages in 2019, as well as time spent in 2019 by IT staff developing, maintaining, and/or upgrading software specifically for MIPS. These were generally fixed costs that did not vary with the number of patients seen. External vendor costs included expenses related to consultants, data analytics firms, and engagement with other health care organizations. For example, some practices reported contracting with consultants to help collect and analyze data or working with a local health system that assisted in quality reporting.

To limit the influence of outliers, time and financial costs were 90% winsorized, setting the bottom 5% to the 5th percentile, the top 5% to the 95th percentile, and then averaging the data. Mean and median time and costs per physician were then calculated for all practices and then for each specialty type and practice size. We used 2-tailed *t* tests with a significance level of .05 to compare mean costs for different types and sizes of physician practices. Data analyses were performed from June 30, 2020, to November 13, 2020, using Stata, release 14.0 (StataCorp LLC). Mean costs are reported in this article; median costs and further details about the methodology can be found in eTables 1 and 2 of the Supplement.

Finally, to mitigate any potential concerns that a physician practice’s strong negative or positive views of MIPS may have influenced a respondent’s estimates of time and cost, 2 researchers (Y.Q. and E.M.O.) independently reviewed transcripts and placed practices into 3 categories: favorable, intermediate, or unfavorable views toward MIPS. There was interrater concordance for 28 of 30 practices; discrepancy on the 2 remaining practices was resolved through discussion. We used *t* tests to evaluate whether per-physician costs differed significantly among practices with favorable, intermediate, and unfavorable views of MIPS (eTable 3 in the Supplement).

## Results

Of 185 physician practices invited, leaders of 30 (9 [30.0%] small primary care, 6 [20.0%] small general surgery, 4 [13.3%] medium primary care , 4 [13.3%] medium general surgery, and 7 [23.3%] large multispecialty) accepted and were interviewed (Table 1). The physician practices in this sample represented all US census regions: the Northeast, 6 practices; the South, 10 practices; the Midwest, 7 practices; and the West, 7 practices (eTable 4 in the Supplement). Of the 30 MIPS-participating practices, 14 (46.7%) practices had participated in a MIPS APM in 2019. The mean percentage of Medicare fee-for-service revenue or patients covered by Medicare Part B was 21.9%.

**Table 1.** Characteristics of Physician Practices Interviewed to Assess Costs of Participating in the Medicare Merit-based Incentive Payment System (MIPS), 2019

Practice type	Practices, No. (%)	Mean size (No. of physicians)^a^	Mean No. of APPs	Medicare share, mean, %^b^	Mean sites	No. of MIPS APM practices
Overall	30 (100)	31.5	17.5	21.9	4.9	14
APM status						
APM	14 (46.7)	23.9	8.4	21.6	4.3	NA
Non–APM	16 (53.3)	38.2	25.4	22.2	5.5	NA
Primary care practice						
Small	9 (30.0)	4.1	2.4	22.4	1.0	6
Medium	4 (13.3)	12.0	3.8	19.4	1.7	2
General surgery practice						
Small	6 (20.0)	5.8	2.0	22.7	1.3	3
Medium	4 (13.3)	19.0	7.8	27.8	9.0	1
Large multispecialty practice	7 (23.3)	107.0	63.6	18.6	13.7	2

Abbreviations: APM, alternative payment model; APPs, advanced practice practitioners; NA, not applicable.

Standard deviations are shown in the Supplement.

^a^ Mean size was defined as the number of individual physicians in the practice.

^b^ Medicare share indicates the proportion of patients covered by or revenue from Medicare fee-for-service.

As shown in the Figure, the mean annual per-physician cost to a physician practice of participating in the MIPS program in 2019 was $12 811 (interquartile range [IQR], $2861-$17 715; eTable 5 in the Supplement). Physician practices that were part of a MIPS APM had mean per-physician costs of $15 410; those that were not part of an APM had mean costs of $10 537. Small and medium primary care practices had mean per-physician costs of $18 466 (IQR, $3240-$36 665) and $13 631 ($7690-$17 684), respectively; small and medium general surgery practices had mean costs of $16 017 (IQR, $10 352-$20 670) and $9690 (IQR, $2248-$11 979), respectively; and large multispecialty practices had mean costs of $4107 (IQR, $1188-$7489). Differences in mean per-physician costs were not statistically significant, except the difference between small general surgery practices and large multispecialty practices (*P* = .01; mean difference, $11 910; 95% CI, $3052-$20 768). See eTable 6 in the Supplement for more details.

**Figure.**
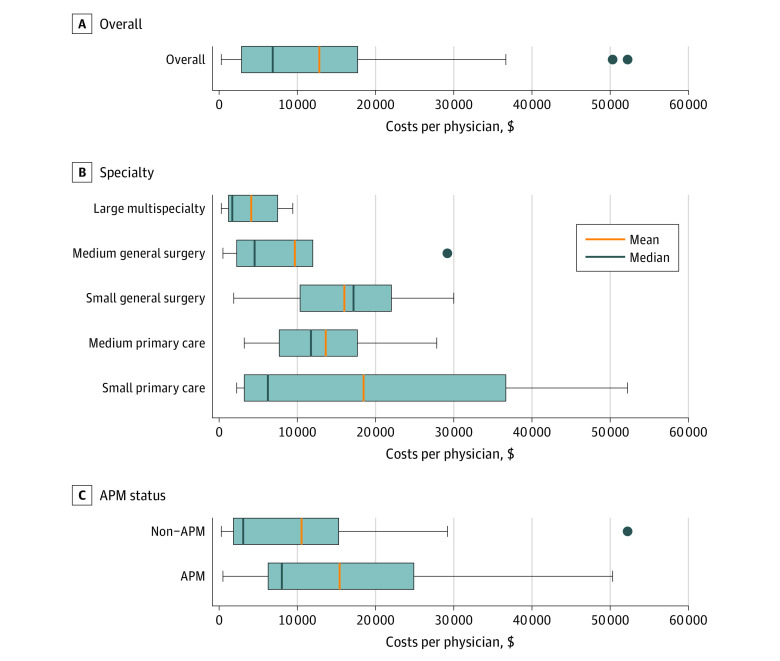
Total Per-Physician Costs of Participating in the Medicare Merit-based Incentive Payment System (MIPS), by Practice Type, 2019 Boxes indicate the interquartile ranges; whiskers, the minimum and maximum values; dots, the outlier physician practices; and APM, alternative payment model. Per-physician costs include MIPS-specific expenses for staff, information technology, and external vendors.

The annual time spent per physician on MIPS-related activities by physicians, clinical staff, and administrators is presented in Table 2. Overall, physicians, clinical staff members, and administrators together spent 201.7 hours annually per physician to participate in the MIPS program in 2019. Nursing staff and MAs spent 99.2 (IQR, 0-163.3) hours per physician to support participation in MIPS; frontline physicians spent 53.6 (IQR, 0-163.3) hours per physician; and executive administrators spent 28.6 (IQR, 3.1-26.7) hours per physician. Other clinicians and staff spent a combined 20.3 (IQR, 0-36.8) hours per physician on MIPS-related activities.

**Table 2.** Annual Time Spent (hours per year) on Activities Related to the Medicare Merit-based Incentive Payment System (MIPS), by Physician Practice Type and Position, 2019

Practice type	Hours per year, by position, mean						
	Administrators		Physicians		APPs	MAs, LPNs, RNs	Total hours
	Executive^a^	Other^b^	Leaders^c^	Other^d^			
Overall	28.6	11.0	0.7	53.6	8.6	99.2	201.7
APM status							
APM	30.6	14.0	1.0	68.2	15.4	104.2	233.4
Non–APM	26.8	8.4	0.3	40.9	2.7	94.8	173.9
Primary care practice							
Small	44.8	18.0	0.7	100.9	17.4	106.1	287.9
Medium	44.0	0	1.4	76.5	2.4	28.4	152.6
General surgery practice							
Small	28.8	1.8	0.4	45.5	10.3	155.1	241.8
Medium	17.8	10.9	1.1	27.8	6.7	97.0	161.4
Large multispecialty practice	4.9	16.4	0.1	1.4	0.6	84.1	107.6

Abbreviations: APM, alternative payment model; APPs, advanced practice practitioners; LPNs, licensed practical nurses; MAs, medical assistants; RN, registered nurses.

Standard deviations are shown in the Supplement.

^a^ Executive administrators are executive-level administrative staff (eg, chief executive officers, practice administrators, staff managers).

^b^ Other administrators are all nonexecutive administrative staff.

^c^ Leaders indicates physician-leaders’ additional time spent on MIPS-related activities beyond time spent on MIPS-related activities as practicing physicians.

^d^ Other physicians indicates practicing physicians’ time spent on MIPS-related activities, excluding physician-leaders’ additional time beyond time spent on MIPS-related activities as practicing physicians.

Table 3 presents the mean per-physician costs for practices by type of staff and other costs. Physician time accounted for a mean cost of $6909 (IQR, $94-$9905) per physician in 2019. When time for MA, LPN, and RNs is converted to dollars, it accounted for $2171 (IQR, $0-$3216) per physician. In large multispecialty practices, the nursing staff and MAs’ time accounted for $2129 (IQR, $0-$3633) per physician, while it accounted for $1739 (IQR, $1428-$2333) in medium general surgery practices and $479 (IQR, $0-$479 [mean and 75th percentile were the same]) in medium primary care practices. Executive administrator time accounted for $1440 (IQR, $201-$1159) per physician and advanced practice clinician (nurse practitioners and physician assistants) time for $431 (IQR, $0-$213) per physician. Average MIPS-related IT costs were also substantial, totaling $1111 (IQR, $64.7-$1716) per physician, while use of external consultants accounted for relatively little cost ($202 per physician; IQR, $0-$357).

**Table 3.** Per-Physician Costs for Participation in the Medicare Merit-based Incentive Payment System (MIPS), by Category of Staff, 2019

Practice type	Category of staff, mean, $^a^									
	Administrators		Physicians		APPs	MAs, LPNs, RNs	IT^b^	External consultant	Costs	
	Executive^c^	Other^d^	Leaders^e^	Other^f^					Other	Total
Overall	1440	303	98	6909	431	2171	1111	202	147	12 811
APM status										
APM	1489	390	137	8545	761	2454	1180	196	257	15 410
Non–APM	1397	227	64	5477	142	1923	1050	207	51	10 537
Primary care practice										
Small	1884	532	78	11 215	900	2581	645	236	393	18 466
Medium	2830	0	188	8889	107	479	944	194	0	13 631
General surgery practice										
Small	1244	101	67	8210	459	3021	2698	217	0	16 017
Medium	1269	185	225	5020	353	1739	640	203	55	9690
Large multispecialty	341	421	25	205	32	2129	712	147	94	4107

Abbreviations: APM, alternative payment model; APPs, advanced practice practitioners; IT, information technology; LPNs, licensed practical nurses; MAs, medical assistants; RN, registered nurses.

Standard deviations are shown in the Supplement.

^a^ Clinician and administrator time were converted to dollars using data on compensation, benefits, and annual time worked from the Medical Group Management Association’s Provider Compensation and Management and Staff Compensation databases.

^b^ IT includes costs related to electronic health records, other software programs, and the IT staff.

^c^ Executive administrators are executive-level administrative staff (eg, chief executive officers, practice administrators, staff managers).

^d^ Other administrators are all nonexecutive administrative staff.

^e^ Leaders indicates physician-leaders’ additional time spent on MIPS-related activities beyond time spent on MIPS-related activities as practicing physicians.

^f^ Other physicians indicate practicing physicians’ time spent on MIPS-related activities, excluding physician-leaders’ additional time beyond time spent on MIPS-related activities as practicing physicians.

## Discussion

Participating in the MIPS program results in substantial financial and time costs for physician practices. We found that, on average, it cost practices $12 811 per physician to participate in MIPS in 2019. We found that physicians themselves spent a considerable amount of time to participate in MIPS. In 2019, physicians spent more than 53 hours per year on MIPS-related activities, which translates to nearly $7000 per physician. If physicians see an average of 4 patients per hour, then these 53 hours could be used to provide care for an additional 212 patients a year—equal to more than a full week’s work for a physician.

According to data from the MGMA, average total revenue (not compensation) for a US general surgeon is $543 562.^24^ Assuming 21.9% revenue from Medicare fee-for-service (the mean proportion in the study sample), a general surgeon receiving a perfect 2018 MIPS score could expect to receive a reward of approximately $2000 in 2020 (eMethods 2 in the Supplement). Potential rewards and penalties will increase to approximately 9% by the 2022 payment year, and the performance score required to avoid a penalty will similarly increase with time. However, it is not clear whether these changes will generate rewards large enough to offset the costs of participation. Despite an increase in the potential incentive size and the minimum performance threshold between the 2019 and 2020 payment years, the maximum reward for MIPS-participating clinicians declined from 1.9% to 1.7%.^3^

Prior work^13^ estimated that physician practices spend approximately 785 hours per physician per year dealing with quality reporting overall. In this study, we find that practices spent more than 201 hours per physician to participate in MIPS alone, and that the time spent by different clinicians and administrators varied widely. MAs, LPNs, and RNs spent the most time on MIPS-related activities, totaling more than 99 hours per physician in 2019. The study interviews suggest that the time was largely spent reviewing medical records, collecting information from patients, and entering data into the EHR. This finding is consistent with other studies that have found that MAs and nursing staff spend more time dealing with payers than do physicians.^19^

Several physician practices appeared to have very high or very low per-physician costs related to MIPS. The 2 practices with very high costs (ie,>$50 000 per physician) had only 2 physicians each, and in 1 case, a part-time RN was hired primarily to assist with MIPS. The practice with the lowest per-physician cost ($279) had more than 270 physicians and may have been able to benefit from economies of scale.

Recent work suggests that value-based payment programs may have a disproportionately negative association with certain practices, including those that are small, rural, independent, or serve a high proportion of patients with low-income.^18,25^ Larger and hospital-affiliated practices tend to have more well-developed reporting infrastructure and more preexisting programs that meet MIPS requirements, and can benefit from better economies of scale.^14,26^ In this study, we find that the costs of participating in MIPS may be greater for small practices and for primary care practices, which would be consistent with prior work on quality reporting^13,19^; however, possibly owing to the relatively small sample size of this study, most differences by size of physician practice and specialty type were not statistically significant. Recent research^2,16^ suggests that physician practices that report through MIPS APMs may receive higher scores than non–APM practices. Although MIPS APMs are intended to simplify reporting for physician practices, per-physician costs did not differ significantly for MIPS APM practices compared with non–APM practices.

Many believe that value-based purchasing programs are important for progress toward a system that rewards quality of care over volume of services.^27,28,29,30^ However, it is increasingly clear that these programs also contribute to administrative burden for clinicians and health care organizations.^31,32^ In a 2019 survey of physician practices,^12^ 84% of practice leaders reported that the US Centers for Medicare & Medicaid Services (CMS) value-based payment reforms had increased administrative burden. Between 2008 and 2018, CMS spent an estimated $1.3 billion developing and maintaining quality measures, producing more than 2200 measures of which nearly 800 have been implemented.^33^ One study found that, compared with Canadian physician practices, US practices spend nearly 4 times as much per physician interacting with payers and US nursing staff spend 10 times as many hours per week doing so.^20^ More generally, administrative costs are a contributor to the outsized cost of the US health care system compared with other high-income countries.^34,35^

The Medicare Payment Advisory Commission has recommended^11,36^ eliminating MIPS because the program may be overly burdensome, may not accurately capture the quality of care, and may divert resources from other worthwhile activities. In recent years, CMS has made several efforts to reduce the administrative burden for physician practices. For example, in 2017, CMS launched the Patients Over Paperwork initiative,^37^ which aims to remove unnecessary regulations and requirements, and the Meaningful Measures Framework,^38^ which attempts to identify high-priority areas for quality measurement. CMS also recently launched a study^39^ to better understand the challenges that physician practices experience when collecting and reporting quality data, but its results are not yet available. In response to the coronavirus disease 2019 pandemic, the CMS has granted MIPS reporting extensions to practices unable to submit data, and in some cases, automatic neutral payment adjustments in 2021 as well.^40^ In the future, CMS could also consider substantially reducing the number of measures used in its quality programs and relying more heavily on claims-based performance measures that do not require physician practices to collect and report data.

### Limitations

This study had several limitations. First, we collected information on costs relevant to only the 2019 performance year. Costs may vary from year to year as MIPS program requirements and practice infrastructures evolve. For example, costs may decrease over time as practices become more familiar with MIPS and can make use of their initial investments in data collection and reporting systems. However, we examined costs during the third year of the program, so practices already had several years of experience with MIPS.

Second, it is possible that physicians and administrators who were more concerned with MIPS costs were more likely to participate. We tried to mitigate this concern by examining whether practices’ views of MIPS were associated with per-physician costs; we did not find statistically significant differences among practices with favorable, intermediate, or unfavorable views (eTable 3 in the Supplement). Furthermore, both the number of participants and the response rate are similar to or higher than those of other qualitative studies evaluating the administrative burden of CMS quality programs,^8,41^ and participants in this study were randomly selected; prior studies have used contractor referrals or convenience samples.^8,41^

Third, in some interviews, respondents provided relatively broad information or a range of costs or time estimates. When ambiguity existed, we consistently used conservative assumptions. A secondary analysis of median costs yields even more conservative estimates—130.3 hours and $6859 per physician per year—and is presented in eTables 1 and 2 in the Supplement.

Fourth, these estimates are based on respondents’ reports, not direct observation. However, these results were broadly consistent with prior studies on the costs to physician practices of quality reporting.^19,20^ Available cost data for other health care organizations (eg, from the American Hospital Association Annual Survey) is also self-reported; by using interviews, instead of surveys, we were able to probe reported costs. Other methods, including time-driven activity-based costing,^42,43^ may yield precise estimates but can be very resource-intensive to perform at scale; for example, a recent study^44^ examining administrative costs in a single academic medical center used 61 interviews to produce estimates.

## Conclusions

In this qualitative study, leaders of physician practices reported substantial time and financial costs of participating in the MIPS program. The attention of policy makers may be warranted to reduce the burden of the MIPS program, particularly given the uncertainty regarding whether it improves quality or outcomes for patients.

## Notes

Supplement 1. **eMethods 1.** Definitions of practice specialties **eMethods 2.** Calculation of maximum positive payment adjustment for average primary care physician in 2020 **eDocument 1.** Interview protocol **eDocument 2.** Invitation letter **eTable 1.** MIPS annual time (hours/y by position) per physician (median) **eTable 2.** MIPS costs domain per physician by category of staff (median) **eTable 3.** Practice attitude ranking **eTable 4.** Practices by US census region **eTable 5.** Total costs per physician of participating in the MIPS program **eTable 6.** Pair-wise comparisons evaluate for differences in the total practice costs per physician of participating in the MIPS program across practice types
