# Supplementary material for: Time and Financial Costs for Physician Practices to Participate in the Medicare Merit-based Incentive Payment System: A Qualitative Study
Source: JAMA Health Forum. 2021 May 14;2(5):e210527. doi: 10.1001/jamahealthforum.2021.0527 (PMC8796897; doi:10.1001/jamahealthforum.2021.0527)
Supplement: Supplement 1. — eMethods 1. Definitions of practice specialties eMethods 2. Calculation of maximum positive payment adjustment for average primary care physician in 2020 eDocument 1. Interview protocol eDocument 2. Invitation letter eTable 1. MIPS annual time (hours/y by position) per physician (median) eTable 2. MIPS costs domain per physician by category of staff (median) eTable 3. Practice attitude ranking eTable 4. Practices by US census region eTable 5. Total costs per physician of participating in the MIPS program eTable 6. Pair-wise comparisons evaluate for differences in the total practice costs per physician of participating in the MIPS program across practice types [file jamahealthforum-e210527-s001.pdf]

Supplemental Online Content

Khullar D, Bond AM, O'Donnell EM, Qian Y, Gans DN, Casalino LP. Time and financial costs for physician practices to participate in the Medicare Merit-based Incentive Payment System: a qualitative study. *JAMA Health Forum*. 2021;2(5):e210527. doi:10.1001/jamahealthforum.2021.0527

eMethods 1. Definitions of practice specialties
eMethods 2. Calculation of maximum positive payment adjustment for average primary care physician in 2020
eDocument 1. Interview protocol
eDocument 2. Invitation letter
eTable 1. MIPS annual time (hours/year by position) per physician (median)
eTable 2. MIPS costs domain per physician by category of staff (median)
eTable 3. Practice attitude ranking
eTable 4. Practices by US census region
eTable 5. Total costs per physician of participating in the MIPS program
eTable 6. Pair-wise comparisons evaluate for differences in the total practice costs per physician of participating in the MIPS program across practice types

This supplemental material has been provided by the authors to give readers additional information about their work.

© 2021 Khullar D et al. *JAMA Health Forum.*

eMethods 1: Definitions of Practice Specialties

Primary care and general surgery practices were defined as having 80% or more physicians in the dominant specialty. Primary care physicians were defined as those practicing family medicine or general internal medicine. General surgery physicians were defined as those practicing general surgery, colorectal surgery, or bariatric surgery. Large multispecialty practices were self-identified as having multiple primary care, medical, and surgical specialties.

© 2021 Khullar D et al. *JAMA Health Forum.*

eMethods 2: Calculation of Maximum Positive Payment Adjustment for Average U.S. General Surgeon in 2020

Assumptions:

1) Total annual revenue of $543,562 (from MGMA DataDive Cost and Revenue Report)
2) Medicare fee-for-service share of 21.9% (mean share in our sample)
3) Maximum payment adjustment of 1.68% in 2020 (publicly available)

Calculation:

$543,562 * 0.219 = $119,040 total Medicare FFS revenue

$119,040 * 0.0168 = $1999.87 positive payment adjustment

© 2021 Khullar D et al. *JAMA Health Forum.*

eDocument 1: Interview Protocol

Description of Project

Hello, my name is [Interviewer Name] from Weill Cornell Medicine. I'm on the line with my colleague [Research Coordinators Name], who will be taking notes. As mentioned when we first contacted you, we would like to understand your practice's financial costs of participating MIPS or MIPS Alternative Payment Models (APMs).

This project is jointly funded by the American Medical Association (AMA) and the Physicians Foundation, but neither was involved in the development of the questions. Please know that your responses will be kept strictly confidential. We will not reveal your name or the name of your group at any point. If you like, we can send you a copy of any articles we publish.

COSTS ASSOCIATED WITH MIPS PARTICIPATION

We want to get a sense of how much it costs physician practices to participate in MIPS. By costs, we mean the expenses associated with staff time, IT infrastructure and software packages, engagement with external vendors or consultants, and any other costs you have incurred. We know it may be hard to unpack the costs of reporting for MIPS versus for other insurers or payment models, but please do the best you can.

I'd like to start by getting a basic understanding of your medical group.

1. The information I have says that your practice is a [specialty] group with [X] physicians and is located in [city, state]. Is this correct?

2. How many different sites does your medical group have?

3. Can you give me a sense of how many other staff are employed by your medical group? By this, I mean nurse practitioners and physician assistants, registered nurses, medical assistants, practice administrators, and any other administrative staff.

4. Approximately what percent of your group's total revenue comes from Medicare?
    a. Probe if necessary: Approximately what percent of your group's patient mix is Medicare patients?

5. Do physicians in your group report to Medicare as individuals, a group, or as a MIPS APM?

6. What mechanism(s) did you use to report data to Medicare for the MIPS program in 2019?

IT Costs and External Vendors

1. Which EHR does your practice use and when did you purchase it?

© 2021 Khullar D et al. *JAMA Health Forum.*

a. To what extent, if any, did you decide to purchase this EHR or update your EHR **because of MIPS**?
    i. Probe: If MIPS partially contributed to your decision, please estimate how likely it is that you would have made the purchase in the absence of MIPS.
    ii. What was the approximate initial cost?
    iii. What is the approximate ongoing cost of operating the EHR?
b. Did your practice purchase any other new software programs **to participate in MIPS**?
    i. Probe: If MIPS partially contributed to your decision, please estimate how likely it is that you would have made the purchase in the absence of MIPS.
    ii. What was the approximate initial cost?
    iii. What is the approximate ongoing cost?

2. Approximately how many hours **annually** (if any) do IT staff in your group spend on **dedicated training programs** in order to participate in MIPS?
    a. Where applicable, please also describe the approximate cost of this training.

3. Approximately how many hours **annually** (if any) do IT staff in your group spend on **dedicated training programs** in order to participate in quality reporting programs, **including but not only for MIPS?**
    a. Where applicable, please also describe the approximate cost of this training.

4. Approximately much time**,** if any, do IT staff spend to help the practice participate or report on various measures, **specifically for MIPS?** If they do spend time on this, please give us your answer in hours per week OR in hours per year.

5. Approximately how much time**,** if any, do IT staff spend to help the practice participate or report on various measures, **including but not only for MIPS?**

6. Does your practice use any outside vendors or consultants to extract or process quality data to submit to CMS for MIPS reporting?
    a. When did you start using this vendor or consultant?
    b. Did you start using this vendor/consultant specifically for MIPS? Or for another program or entity?
        i. Probe: If MIPS partially contributed to your decision, please estimate how likely it is that you would have done so in the absence of MIPS.
    c. What was the approximate initial cost of the vendor/consultant?
    d. What is the approximate ongoing cost of working with this vendor/consultant?

Costs to your group of other forms of activity related to MIPS:

7. In order to participate in the MIPS program, have you hired any new staff members?
    a. If so, how many and in which positions?
    b. Approximately when were they hired?
    c. Approximately what % of their time do they spend on MIPS-related activities?

We are interested in the time that members of your medical group – both leadership and rank-and-file spend on activities related to MIPS.

We are interested in any and all of the following types of people, or others if we are missing any that do work for your medical group related to MIPS.

a. *Physician-leaders*
b. *Practicing physicians*
c. *Non-physician administrative leaders*
d. *Other administrative staff (e.g., information technology (IT), billing and coding, or medical records staff)*
e. *Nurse practitioners and/or Physician Assistants*
f. *Registered Nurses*
g. *Medical Assistants, LPNs, or LVNs*

Medical groups might engage in several types of activity to deal with MIPS. Now we are going to ask you about these activities:

8. *Tracking and understanding the quality measures used by MIPS, and tracking and understanding any other MIPS program requirements.*

    Who does this work in your group (e.g. physician leaders, administrative leaders . . .)?

    Approximately how much time do they spend on this activity? We would like to know how many [ask for each type of person they have mentioned] hours per week or per year, whichever is easier for you to estimate, each person spends.

    Is there anyone else in your group who spends time tracking and understanding MIPS? [If so, as who, type of person, how much time. When we suspect that certain types of people may engage in these activities, we can ask specifically – they will get the idea.]

9. *Participating in or conducting training programs intended to help your group deal with MIPS.*
    [Ask same questions as in question #8.]

10. *Entering information into the EHR specifically for MIPS*
    [Ask same questions as in question #8.]

11. *Collecting quality data on paper or electronically <u>specifically for MIPS, and/or transmitting this data to CMS</u>*
    [Ask same questions as in question #8.]

© 2021 Khullar D et al. *JAMA Health Forum.*

16. *Creating and/or participating* ***in quality improvement efforts*** ***<u>specifically for MIPS?</u>*** *(If necessary, can give examples: extended hours, population health management activities, integrating behavioral healthcare.*

    [Ask same questions as in question 8]

17. *Creating and/or participating* ***in quality improvement efforts*** **(<u>including but not only for MIPS</u>)?**

    [Ask same questions as in question #8.]

18. **Entering information into the EHR <u>specifically for MIPS?</u>**

    [Ask same questions as in question #8.]

19. Entering **information into the EHR for quality reporting in general (<u>including but not only for MIPS</u>)?**

    [Ask same questions as in question #8.]

20. Collecting **quality data on paper or electronically <u>specifically for transmission MIPS</u>**?

    [Ask same questions as in question #8.]

21. Collecting **quality data on paper or electronically and transmitting them for quality reporting in general (<u>including but not only for MIPS)</u>?**

    [Ask same questions as in question #8.]

22. Other than the activities we have discussed, are there any other activities that members of your medical group do that are related to MIPS?
    [Ask same questions as in question #8.]

23. Are there any other costs you have incurred while dealing with MIPS that we haven't discussed?

24. Is there anything that we haven't asked you about that you would like to add?

If you would like a copy of the articles we publish from these interviews, please let us know the best email to send them to.

PRACTICE VIEWS ON MIPS

We'd also like to get a sense of your general views on the MIPS program.

© 2021 Khullar D et al. *JAMA Health Forum.*

1. Has participating in the MIPS program led you to consider merging with or selling to a larger health care organization? If so, why?

2. As you know, MIPS allows practices to select 6 quality metrics out of a list of several hundred to report on. How did you decide which quality metrics you would report?

3. In your opinion, how could the MIPS program be improved?

4. In what ways do you feel MIPS has helped or hindered your efforts to provide high quality care to your patients?

© 2021 Khullar D et al. *JAMA Health Forum.*

eDocument 2: Pre-interview Letter

INITIAL EMAIL INVITATION

Subject: Understanding the Costs to Practices of Participating in MIPS

Dear [Invitee],

Since 2017, Medicare has used the Merit-based Incentive Payment System (MIPS) to collect quality and cost information from physicians and medical groups. MGMA is collaborating with Weill Cornell Medicine to study the costs to practices associated with participating in MIPS.

I am asking if you will participate in a telephone interview to share your insights about MIPS and provide information on the financial costs of participating in MIPS. The identity of participants and all information collected in our study is strictly confidential and the study will not identify any specific individuals or practices.

Our findings will be submitted for publication in a peer-reviewed journal and will also be provided to the American Medical Association and the Physicians Foundation who are funding the research. Our team has published numerous articles in the *New England Journal of Medicine*, *JAMA*, and other medical and health policy journals.

Prior to the interview we will send you a list of questions we will ask. We recognize that your time is extremely valuable, so we are offering a **$X honorarium for your participation.**

If you are willing to participate, please respond to my message. I will then send additional information on the study and I will provide your contact information to our research team to schedule an interview.

If you have questions regarding the study, please contact me directly by email or you can phone me at MGMA, XXX-XXX-XXXX or XXXX@XXXX.com.

Thank you for your participation in this important study.

XXX

© 2021 Khullar D et al. *JAMA Health Forum.*

eTable 1. MIPS Annual Time (hours/year by position) Per Physician (Median)

**Practice Type**	**Hours Spent on MIPS by Position per Physician per Year (Median)**						
	**Administrators**		**Physicians**		**APPs**	**MAs, LPNs, RNs**	**Total Hours**
	**Executive**[a]	**Other**[b]	**Leaders**[c]	**Other**[d]			
**Overall**	8.7	0.0	0.0	8.0	0.0	66.8	130.3
**APM Status**							
APM	7.1	0.0	0.0	16.7	0.0	89.5	187.4
Non-APM	9.3	0.3	0.0	4.4	0.0	31.6	108.2
**Specialty**							
Small Primary Care	19.0	5.0	0.0	12.7	3.0	59.2	105.1
Medium Primary Care	18.4	0.0	0.0	62.8	0.0	0.0	142.8
Small General Surgery	20.8	0.0	0.0	45.5	0.0	157.5	250.8
Medium General Surgery	8.5	0.0	0.1	1.7	1.6	112.4	145.4
Large Multispecialty	4.8	3.4	0.0	0.4	0.0	0.0	45.3

Abbreviations: MIPS, Merit-Based Incentive Payment System; APM, Alternative Payment Model; APPs, advanced practice practitioners; MAs, medical assistants; LPNs, licensed practical nurses; RN, registered nurses. The mean and standard deviation for each variable are shown in the supplement.

[a] Executive administrators include the administrative staff at executive-level positions such as chief executive officer, practice administrators, and staff manager.

[b] Other administrators include administrative staff other than executive administrators.

[c] Leaders indicates physician leaders' additional time spent on MIPS-related activities beyond their time spent on MIPS-related activities as practicing physicians.

[d] Other physicians indicate practicing physicians' time spent on MIPS-related activities, excluding physician leaders additional time beyond their time spent on MIPS-related activities as practicing physicians.

© 2021 Khullar D et al. *JAMA Health Forum.*

eTable 2. MIPS Costs Domain per Physician by Category of Staff (Median)

Practice Type	MIPS Costs per Physician per Year by Type of Staff (Median)									
	Administrators		Physicians		APPs	MAs, LPNs, RNs	Information Technology[e]	External Consultant	Other Costs	Total Costs
	Executive[a]	Other[b]	Leaders[c]	Other[d]						
**Overall**	$534	$0	$0	$917	$0	$1,240	$455	$0	$0	$6,859
**APM Status**										
APM	$381	$0	$0	$1,782	$0	$1,670	$704	$0	$0	$8,025
Non-APM	$599	$11	$0	$624	$0	$582	$324	$0	$0	$3,078
**Specialty**										
Small Primary Care	$823	$85	$0	$1,356	$195	$1,056	$350	$0	$0	$6,238
Medium Primary Care	$1,626	$0	$0	$7,352	$0	$0	$980	$92	$0	$11,743
Small General Surgery	$907	$0	$0	$8,209	$0	$2,808	$2,938	$0	$0	$17,181
Medium General Surgery	$514	$0	$13	$312	$117	$2,022	$417	$31	$0	$4,538
Large Multispecialty	$278	$85	$0	$77	$0	$0	$108	$153	$0	$1,690

© 2021 Khullar D et al. *JAMA Health Forum.*

Abbreviations: MIPS, Merit-Based Incentive Payment System; APM, Alternative Payment Model; APPs, advanced practice practitioners; MAs, medical assistants; LPNs, licensed practical nurses; RN, registered nurses. We converted clinician and administrator time in the TABLE 3 to dollars using data on compensation, benefits, and annual time worked from the MGMA. Additional details on the cost calculation methods can be found in the manuscript, the Analysis section. The mean and standard deviation for each variable are shown in the supplement.

[a] Executive administrators include the admin staff at the executive-level positions such as chief executive officer, practice administrators, staff manager…etc.

[b] Other administrators include administrative staff other than executive administrators.

[c] Leaders indicates physician leaders' additional time spent on MIPS-related activities beyond their time spent on MIPS-related activities as practicing physicians.

[d] Other physicians indicate practicing physicians' time spent on MIPS-related activities, excluding physician leaders additional time beyond their time spent on MIPS-related activities as practicing physicians.

[e] Information Technology (IT) includes EHR related costs, other software program costs and the IT staff costs.

© 2021 Khullar D et al. *JAMA Health Forum.*

eTable 3. Practice Attitude Ranking

Practice Attitude	Practice Name	Practice Type	Total Practice Costs per Physician[a]	Mean[b]	Median[c]
Positive	Practice 1	Small Primary Care	$3,240	$8,991	$7,619
	Practice 2	Small General Surgery	$16,113		
	Practice 3	Large Multispecialty	$7,619		
Intermediate	Practice 4	Small Primary Care	$5,953	$12,338	$6,300
	Practice 5	Small Primary Care	$2,941		
	Practice 6	Small Primary Care	$36,665		
	Practice 7	Small Primary Care	$50,303		
	Practice 8	Small Primary Care	$52,211		
	Practice 9	Small Primary Care	$6,359		
	Practice 10	Medium Primary Care	$27,823		
	Practice 11	Medium Primary Care	$9,182		
	Practice 12	Medium Primary Care	$14,304		
	Practice 13	Medium Primary Care	$3,216		
	Practice 14	Small General Surgery	$1,869		
	Practice 15	Small General Surgery	$21,477		
	Practice 16	Small General Surgery	$8,432		
	Practice 17	Medium General Surgery	$489		
	Practice 18	Medium General Surgery	$6,241		
	Practice 19	Medium General Surgery	$2,835		
	Practice 20	Large Multispecialty	$1,248		
	Practice 21	Large Multispecialty	$279		

© 2021 Khullar D et al. *JAMA Health Forum.*

	Practice 22	Large Multispecialty	$9,426		
	Practice 23	Large Multispecialty	$7,360		
	Practice 24	Large Multispecialty	$1,690		
	Practice 25	Large Multispecialty	$1,128		
Negative	Practice 26	Small Primary Care	$6,239	$17,185	$18,249
	Practice 27	Small Primary Care	$2,283		
	Practice 28	Small General Surgery	$29,960		
	Practice 29	Small General Surgery	$18,249		
	Practice 30	Medium General Surgery	$29,194		

The pair-wise t-test was performed to determine if the mean total practice costs per physician differed significantly between any two of the three attitude groups (positive, intermediate, and negative). All differences are not significant at the 0.05 level.

[a] Total practice costs per physician indicates per physician cost to practices of participating in the MIPS program in 2019. The cost includes staff expenses, IT costs, and external vendor costs specifically for MIPS. Additional details on the cost definition and calculation methods can be found in the manuscript Analysis section.

[b,c] The mean and median cost for each attitude group (positive, intermediate, and negative) were calculated.

© 2021 Khullar D et al. *JAMA Health Forum.*

eTable 4. Practices by U.S. Census Region

Practice Type	Northeast	South	Midwest	West
**Overall**	6	10	7	7
**Small Primary Care**	1	5	1	2
**Medium Primary Care**	1	1	2	0
**Small General Surgery**	3	1	0	2
**Medium General Surgery**	1	1	1	1
**Large Multispecialty**	0	2	3	2

© 2021 Khullar D et al. *JAMA Health Forum.*

eTable 5. Total Costs per Physician of Participating in the MIPS Program

Practice Type	Costs per Physician[a]			
	Mean	Median	25th Percentile	75th Percentile
**Overall**	$12,811	$6,859	$2,861	$17,715
**APM Status**				
APM	$15,410	$8,025	$6,269	$24,896
Non-APM	$10,537	$3,078	$1,825	$15,290
**Specialty**				
Small Primary Care	$18,466	$6,238	$3,240	$36,665
Medium Primary Care	$13,631	$11,743	$7,690	$17,684
Small General Surgery	$16,017	$17,181	$10,352	$20,670
Medium General Surgery	$9,690	$4,538	$2,248	$11,979
Large Multispecialty	$4,107	$1,690	$1,188	$7,489

Abbreviations: MIPS, Merit-Based Incentive Payment System; APM, Alternative Payment Model.
[a] Costs per physician indicates per physician cost to practices of participating in the MIPS program in 2019. The cost includes staff expenses, IT costs, and external vendor costs specifically for MIPS. Additional details on the cost definition and calculation methods can be found in the manuscript Analysis section.

eTable 6. Pair-wise Comparisons Evaluate for Differences in the Total Practice Costs per Physician of Participating in the MIPS Program across Practice Types

Practice Type	Small Primary Care[a]	Medium Primary Care[b]	Small General Surgery[c]	Medium General Surgery[d]	Large Multispeci alty[e]	MIPS APM[f]
**Small Primary Care**	NA	0.681	0.799	0.470	0.104	NA
**Medium Primary Care**	0.681	NA	0.724	0.657	0.053	NA
**Small General Surgery**	0.799	0.724	NA	0.409	0.013	NA
**Medium General Surgery**	0.470	0.657	0.409	NA	0.308	NA
**Large Multispecialty**	0.104	0.053	0.013	0.308	NA	NA
**Non-APM**	NA	NA	NA	NA	NA	0.365

Abbreviations: MIPS, Merit-Based Incentive Payment System; APM, Alternative Payment Model; APPs, NA, not applicable.
Significance tests were performed using two-sample T-Test. All differences were not significant at the 5% significance level, except for the difference in the cost per physician between small general surgery practice and large multispecialty practice (*P*=0.013).
[a] Small primary care indicates the P-values obtained from the comparisons for differences in the total practice costs per physician of participating in the MIPS between small primary care practices and each of the other practice types.
[b] Medium primary care indicates the P-values obtained from the comparisons for differences in the total practice costs per physician of participating in the MIPS between medium primary care practices and each of the other practice types.
[c] Small general surgery indicates the P-values obtained from the comparisons for differences in the total practice costs per physician of participating in the MIPS between small general surgery practices and each of the other practice types.
[d] Medium general surgery indicates the P-values obtained from the comparisons for differences in the total practice costs per physician of participating in the MIPS between medium general surgery practices and each of the other practice types.
[e] Large multispecialty indicates the P-values obtained from the comparisons for differences in the total practice costs per physician of participating in the MIPS between large multispecialty practices and each of the other practice types.
[f] MIPS APM indicates the P-values obtained from the comparisons for differences in the total practice costs per physician of participating in the MIPS between APM practices and Non-APM practices.
